# Unique Features of Melanoma Risk and Diagnosis in Red-Haired Populations

**DOI:** 10.3390/ijms27177765

**Published:** 2026-08-30

**Authors:** Kiran R. Ebrahimi, Stephen M. Ostrowski, David E. Fisher

**Affiliations:** Cutaneous Biology Research Center, Department of Dermatology, Massachusetts General Hospital, Harvard Medical School, Boston 02129, MA, USA; kebrahimi@mgh.harvard.edu (K.R.E.); sostrowski@mgh.harvard.edu (S.M.O.)

**Keywords:** melanoma, *MC1R*, red-hair color phenotype, melanin, eumelanin, pheomelanin, amelanotic melanoma, nevi, skin cancer screening

## Abstract

Cutaneous melanoma is the most lethal skin cancer, and risk is strongly shaped by pigmentary phenotype. The red-hair color phenotype, marked by red hair, fair skin, freckling, and poor tanning, carries elevated melanoma risk driven largely by loss-of-function variants in the melanocortin 1 receptor gene (*MC1R*). This risk is not explained by reduced ultraviolet protection alone. Impaired *MC1R* signaling shifts melanogenesis from photoprotective eumelanin toward pheomelanin, a pigment associated with oxidative stress and partly ultraviolet-independent melanomagenesis. *MC1R* may also influence melanoma susceptibility through pigment-independent effects on DNA damage responses, repair signaling, and genomic stability. These mechanisms support investigation of *MC1R* genotype, visible phenotype, nevus burden, pigment chemistry, and imaging-derived lesion metrics as complementary tools for risk stratification. Diagnosis is also distinctive in this population. Amelanotic and hypomelanotic melanomas are associated with the red-hair color phenotype, and their low pigmentary contrast may delay recognition and contribute to diagnosis at a more advanced stage. This review integrates genetic, molecular, biomarker, diagnostic, and therapeutic literature specific to red-haired populations. We argue that elevated biological susceptibility and diagnostic difficulty compound one another, and we outline priorities for *MC1R*-informed surveillance, imaging adapted to pigment-poor disease, pharmacologic modulation of *MC1R*-related pathways, and prospective risk models integrating genotype, phenotype, nevus burden, pigment biology, and imaging.

## 1. Introduction

Cutaneous melanoma accounts for a minority of skin cancer diagnoses but a large majority of skin cancer mortality. Although advances in immunotherapy have improved outcomes for advanced disease, prognosis remains strongly dependent on the stage at which the primary lesion is detected and excised. Melanoma survival is therefore shaped by both the biological conditions that allow malignant transformation and the clinical systems by which early lesions are recognized. Both vary across populations defined by pigmentary phenotype [1,2,3,4].

Red hair, fair skin, freckling, and poor tanning ability have long been associated with increased melanoma risk [5,6,7,8]. These traits cluster in the red-hair color (RHC) phenotype, which is driven primarily by loss-of-function mutations in the melanocortin 1 receptor gene, *MC1R* [9]. *MC1R* inactivation leads to a decrease in photoprotective eumelanin, but increased melanoma risk in red-haired populations is not explained by reduced ultraviolet shielding alone. Loss of *MC1R* signaling shifts melanogenesis away from photoprotective eumelanin and toward pheomelanin, a pigment that provides weaker ultraviolet protection and contributes to oxidative damage even without direct ultraviolet exposure [10]. *MC1R* may also influence DNA repair and genomic stability through mechanisms that are separable from pigment production [11,12]. These findings suggest that red hair marks a broad biological state of melanoma vulnerability.

This biology has implications for risk stratification, prevention, and early detection. *MC1R* genotype, visible pigmentary phenotype, nevus burden, family history, prior melanoma, and ultraviolet exposure provide complementary but incomplete measures of susceptibility. Biochemical measures of melanogenesis and pigment chemistry may be useful in research settings but they are not yet validated for routine melanoma susceptibility prediction. At the same time, pharmacologic strategies that act downstream of *MC1R* or modify receptor signaling, including cAMP activation, salt-inducible kinase inhibition, and modulation of *MC1R* palmitoylation, suggest that some aspects of *MC1R*-associated vulnerability may be modifiable [13,14,15,16]. These approaches remain preclinical or investigational, but they illustrate how inherited pigmentation biology may inform future preventive strategies.

The clinical problem is compounded by diagnostic challenges. Amelanotic and hypomelanotic melanomas are highly associated with the red-hair color phenotype and can delay diagnosis [17]. Lesions with little or no pigment may fail to meet classic visual expectations for melanoma, delaying recognition by patients and clinicians. A group with elevated melanoma risk may therefore also face a higher likelihood that some melanomas will be visually subtle or atypical [18,19]. This challenge motivates interest in surveillance strategies that extend beyond standard visual inspection, including total-body photography, sequential digital dermoscopy, three-dimensional imaging, reflectance confocal microscopy, optical coherence tomography, multispectral or hyperspectral imaging, and carefully validated artificial intelligence-assisted triage.

This review examines the connection between *MC1R* biology, melanoma susceptibility, biomarkers, and diagnosis in red-haired populations. We first discuss *MC1R* signaling and the biochemical switch between eumelanin and pheomelanin production and then consider the genetic architecture of the red-hair color phenotype and the incomplete relationship between *MC1R* genotype and visible pigmentation. We next evaluate pigment-dependent and pigment-independent routes to melanoma risk, including ultraviolet-mediated DNA damage, pheomelanin-associated oxidative stress, non-pigment roles of *MC1R*, and emerging preventive strategies [10,20]. Finally, we examine nevus burden, biomarker-informed surveillance, advanced imaging, and the diagnostic challenges posed by amelanotic and hypomelanotic melanoma [17,21,22].

This review was informed by literature searches conducted in PubMed and Google Scholar on 1 May 2026, and updated on 25 May 2026. The search included English-language publications from 1975 through 2026. Search strings included: (“*MC1R*” OR “melanocortin 1 receptor”) AND (“red hair” OR “red-hair phenotype” OR “red hair color phenotype”) AND (“melanoma” OR “melanoma risk”); (“*MC1R*” OR “red hair”) AND (“nevus” OR “nevi” OR “nevus count”); and (“*MC1R*” OR “red hair”) AND (“amelanotic melanoma” OR “hypomelanotic melanoma” OR “melanoma pigmentation”). Additional searches combined *MC1R* or red-hair-related terms with “pheomelanin,” “eumelanin,” “oxidative stress,” “DNA damage,” “melanoma detection,” “imaging,” “surveillance,” and “melanoma prevention.”

Peer-reviewed original human studies were considered when they evaluated *MC1R* genotype or red-hair-associated pigmentation traits in relation to melanoma susceptibility, nevus count, melanoma pigmentation, diagnosis, surveillance, or clinical outcomes. Experimental studies were considered when they examined *MC1R* signaling, melanogenesis, oxidative stress, DNA-damage responses, or preventive strategies relevant to the review. Review articles were used to identify additional primary studies and provide background context. Editorials, conference abstracts, non-peer-reviewed reports, and studies that did not address the review focus were excluded. Reference lists of included articles and relevant reviews were also examined to identify additional publications.

## 2. *MC1R* Signaling and the Eumelanin–Pheomelanin Switch

### 2.1. The Receptor cAMP Cascade and Control of Pigment Type

The melanocortin 1 receptor is a G protein-coupled receptor present at the melanocyte cell surface that governs the balance between the two principal classes of melanin pigment, eumelanin and pheomelanin. Its primary physiological agonist is alpha-melanocyte-stimulating hormone (α-MSH). In response to UV-induced DNA damage, p53 activation in keratinocytes induces transcription of proopiomelanocortin (*POMC*), and the resulting prohormone is proteolytically cleaved to yield multiple peptides, including α-MSH [23].

**Figure 1:**
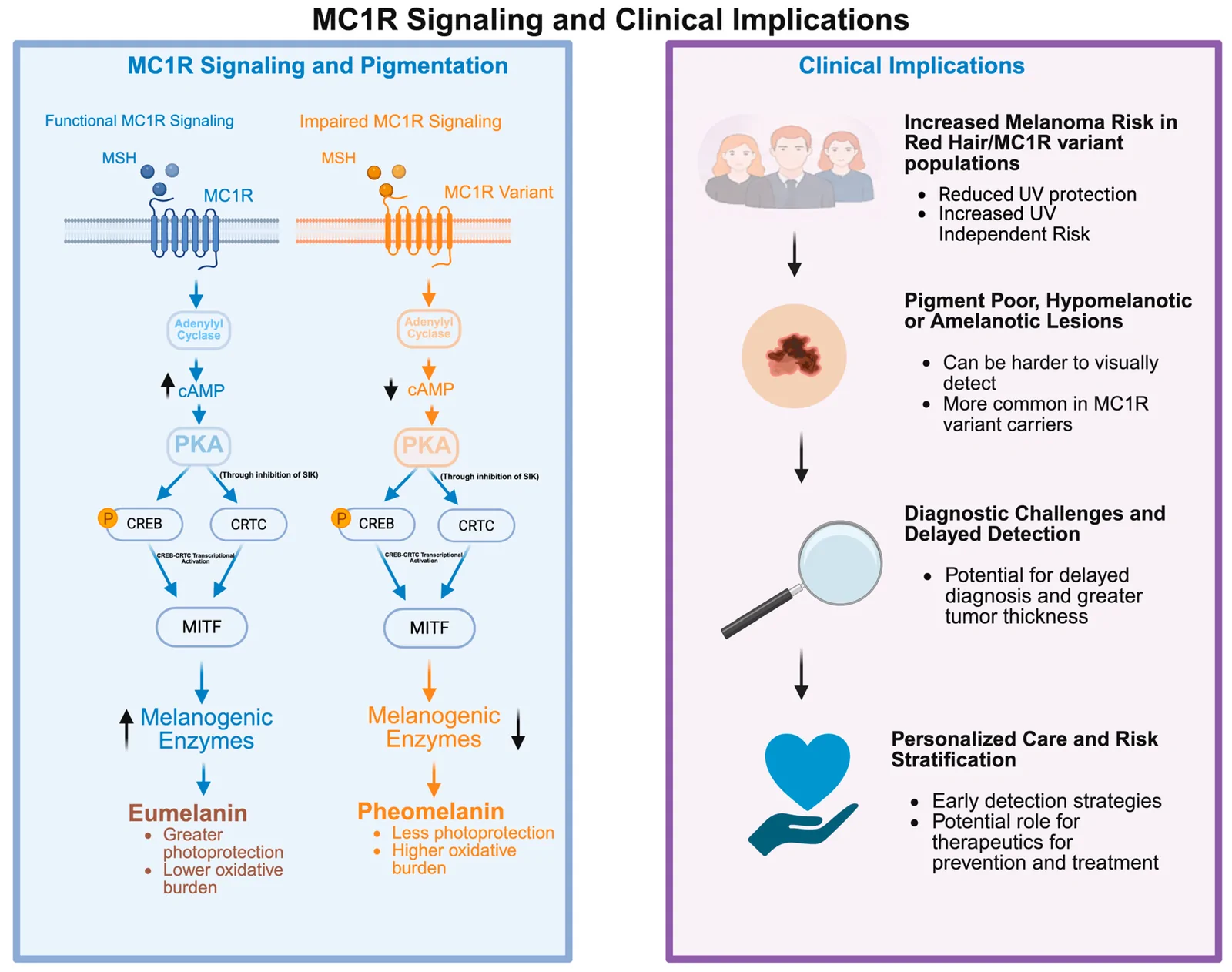
Graphical Presentation of *MC1R* Pathway and Clinical Implications (Created in BioRender. Ebrahimi, K. (2026). BioRender.com/z7ff7rg).

The binding of α-MSH stabilizes the active receptor conformation of *MC1R*, which couples through the stimulatory G protein to adenylyl cyclase and raises intracellular 3′,5’-cyclic adenosine monophosphate (cAMP). Elevated cAMP activates protein kinase A (PKA), which directs both activating phosphorylation of CREB1 and inhibitory phosphorylation of salt-inducible kinase (SIK) to remove SIK-mediated repression of CRTC [14]. CREB and CRTC are recruited to the promoter of the M isoform of microphthalmia-associated transcription factor (MITF-M), the master regulator of the melanocyte lineage that controls expression of melanocyte differentiation and melanogenic enzyme genes [24,25]. Through these targets, *MC1R* signaling increases melanocytes’ capacity to synthesize eumelanin, the brown-black pigment that absorbs ultraviolet radiation and limits photodamage (Figure 1) [13,26,27].

Pigment type is also influenced by antagonistic signaling. Agouti signaling protein (ASIP) can oppose *MC1R* activity and reduce cAMP signaling, shifting melanogenesis away from eumelanin and toward pheomelanin [28]. ASIP has been associated with red hair in GWAS studies, while rare germline activating mutations of ASIP result in the red-hair color phenotype in combination with obesity due to ASIP modulation of neuronal MC4R signaling [29]. Loss-of-function *MC1R* variants in both mouse and human reduce receptor activation and are associated with red hair/fur [30,31,32].

### 2.2. Eumelanin and Pheomelanin Synthesis and Photoprotection

Eumelanin and pheomelanin arise from a shared melanogenic pathway but diverge after the formation of dopaquinone. Tyrosinase catalyzes the rate-limiting oxidation of tyrosine to dopaquinone, creating a chemically reactive branch point in melanin synthesis. When sulfhydryl compounds such as cysteine and glutathione are abundant, dopaquinone reacts with these thiols to form cysteinyldopa and glutathionyldopa intermediates, which are subsequently oxidized and polymerized into pheomelanin [33]. This pathway yields a sulfur-containing red-yellow pigment enriched in red hair and fair skin. When thiol availability is lower, dopaquinone instead undergoes intramolecular cyclization to cyclodopa, followed by oxidation to dopachrome and further reactions that generate eumelanin. This pathway yields the brown-black pigment characteristic of darker pigmentation [34,35].

The two pigments differ substantially in their physical and redox properties. Eumelanin is a broadband absorber of ultraviolet and visible radiation and can dissipate absorbed energy in ways that limit photochemical damage. It can also participate in redox buffering, helping to neutralize reactive oxygen species (ROS) [36,37]. Surprisingly, the absolute UV shielding provided by eumelanin is rather low (equating to a sun protection factor/SPF of 2–4), suggesting that ROS buffering or other mechanisms may play a key role in eumelanin-mediated UV protection [38,39].

## 3. Genetic Architecture of the Red-Hair Color Phenotype

*MC1R* is among the most polymorphic pigmentation genes in populations of European ancestry, and individual variants differ substantially in their effects on receptor function, pigmentary phenotype, and melanoma risk [9,40,41]. A subset of variants including D84E, R151C, R160W, and D294H is conventionally classified as high penetrance red hair color variants, or “R” alleles, because they are strongly associated with red hair and cause substantial impairment of *MC1R* function [42,43]. A second group of lower penetrance variants, often designated “r” alleles, show weaker associations with red hair. These alleles may contribute to fair pigmentation or melanoma risk but are less consistently associated with overt red hair.

The red hair phenotype depends on both the identity and combination of *MC1R* alleles. Red hair is most strongly associated with genotypes containing two functionally consequential alleles, particularly R/R and some R/r combinations. However, *MC1R*-associated pigmentation does not follow a simple recessive model. Individuals carrying a single R allele, two r alleles, or other combinations may exhibit components of the broader red-hair color phenotype, including fair skin, freckling, poor tanning, and increased sun sensitivity, without having visibly red hair. Large-scale analysis of hair color in the UK Biobank demonstrated approximately 57% of individuals carrying two *MC1R* “R” variants reported red hair [20]. This incomplete penetrance indicates modification by genetic background, by allele combinations and allele-dependent penetrance [43,44].

A melanoma risk model based only on visible red hair will therefore miss some *MC1R* variant carriers whose pigmentation appears less distinctive, while a model based only on genotype may include individuals whose clinical phenotype does not immediately suggest high risk. Future work should determine how best to integrate *MC1R* genotype, visible pigmentation, and polygenic background into melanoma risk prediction [45,46].

## 4. Pigment-Dependent and Pigment-Independent Routes to Melanoma

### 4.1. Reduced Photoprotection and UV Damage

The longest-standing explanation for the elevated melanoma risk associated with the RHC phenotype is reduced photoprotection. Eumelanin absorbs and dissipates ultraviolet radiation more efficiently than pheomelanin, limiting the amount of UV energy that reaches nuclear DNA in epidermal melanocytes. In eumelanin-poor skin, this shielding is diminished, increasing the likelihood of UV-induced DNA photoproducts such as cyclobutane pyrimidine dimers (CPDs) and 6-4 photoproducts. These lesions, if unrepaired or misrepaired, can give rise to the C to T transitions and CC to TT substitutions characteristic of the ultraviolet mutagenic signature in melanoma [47,48].

Pheomelanin may interact with UV by other mechanisms to increase melanoma risk. Under ultraviolet exposure, pheomelanin can behave as a photosensitizer and pro-oxidant, promoting reactive oxygen species formation and consuming antioxidant reserves. For a given dose of ultraviolet exposure, a pheomelanin-rich melanocyte may therefore experience both greater direct photochemical injury and greater oxidative stress than a eumelanin-rich melanocyte. The UV-dependent route to melanoma in red-haired populations is thus best understood as a combination of reduced optical shielding and pigment chemistry that can amplify damage once irradiation occurs [49,50,51].

### 4.2. Pheomelanin-Driven Oxidative Stress

Existing evidence suggests that pheomelanin can contribute to melanomagenesis even without ultraviolet exposure. UV-independent processes in melanoma have long been suggested, in part due to clinical evidence that melanoma commonly forms on relatively sun-protected sites and the fact that most driver mutations in melanoma (*BRAF^V600E^*, *NRAS^Q61^*) do not have “UV signature” nucleotide changes [52,53]. Direct experimental support came from a mouse model carrying an inactivating *MC1R* mutation (*MC1R^e/e^*) and having orange hair, analogous to the human RHC phenotype. When *Braf^V600E^* was expressed in melanocytes in this background, invasive melanomas arose at substantial frequency even in the absence of UV exposure, but not in *MC1R*-intact black-furred mice. Introducing a tyrosinase-null albino background (*MC1R^e/e^, Tyr^c/c^, Braf^V600E^*), which abolishes pigment synthesis, including pheomelanin, markedly reduced melanoma formation despite the presence of the same oncogenic driver. Pheomelanin-bearing skin also showed increased markers of oxidative DNA and lipid damage compared with pigment-deficient skin. Together, these findings support a model in which pheomelanin synthesis itself can increase oxidative stress and promote melanoma development independently of direct ultraviolet irradiation [10].

While these mechanistic studies were done in animal models, human epidemiologic data support the relevance of this pathway beyond mouse models. In a hospital-based case–control study of melanoma patients and controls, *MC1R* variant carriers remained at increased melanoma risk even after adjustment for prior sunburns and dermatologist-assessed signs of actinic damage. Individuals carrying two or more *MC1R* variants had approximately a twofold higher melanoma risk than wild-type carriers after controlling for these measures of UV exposure. These findings strengthen the case that *MC1R*-associated risk cannot be explained solely by measured ultraviolet exposure and reflects intrinsic pigmentary or signaling mechanisms that persist independently of sun damage [54,55].

Sequencing of human melanomas shows that those with germline *MC1R* R alleles are associated with a 42% higher somatic mutational burden, including an increased burden of C to T transitions linked to ultraviolet exposure [56]. The magnitude of this increase has been compared with the mutational burden associated with an additional two decades of age. These data strengthen the case that *MC1R*-related pigmentation biology affects the amount and character of DNA damage that accumulates in melanoma, while also leaving open how much of this effect is mediated by UV-dependent and UV-independent processes. It is additionally important to consider that many cutaneous melanomas arise in non-red-haired individuals who have very light skin (epidermal) pigmentation, likely mimicking the paucity of eumelanin photoprotection despite stronger *MC1R* signaling within hair follicles.

A remaining challenge is to determine how pheomelanin chemistry produces oxidative stress in intact biological systems. The relative contributions of ultraviolet absorption, ROS generation, and antioxidant depletion are difficult to separate experimentally, especially because these processes may occur simultaneously within the melanosome and surrounding cytoplasm. It also remains unclear whether antioxidant depletion reflects cysteine consumption during pheomelanin synthesis, direct reactions between antioxidants and pheomelanin-derived ROS, or both. Although it has been shown that melanin and UV-induced ROS can induce CPD formation, and pheomelanin is well supported as a source of ROS after ultraviolet exposure, it remains incompletely understood whether pheomelanin-induced ROS also contributes to CPD formation [57,58].

### 4.3. *MC1R* Functions Beyond Pigment

*MC1R* exhibits signaling functions distinct from pigmentation, and these may play a role in melanoma risk. Early studies showed that α-MSH signaling reduces ultraviolet-induced apoptosis and CPD accumulation in human melanocytes, and subsequent work linked this protection to enhanced repair of UV photoproducts through *MC1R*-dependent signaling [12,59]. Red-hair-associated *MC1R* variants blunt these responses, suggesting that loss of receptor function may impair the melanocyte DNA damage response in addition to shifting pigment production toward pheomelanin. *MC1R* signaling has also been implicated in chromosome stability and centromeric integrity in melanocytes, suggesting that receptor loss may promote melanoma susceptibility not only by diminishing eumelanin-associated photoprotection but also by weakening broader melanin independent mechanisms of genome integrity/maintenance [11,60,61,62].

It is also unclear how some of these protective effects are coordinated at the molecular level. Some key downstream steps linking *MC1R* signaling to DNA repair have not been fully resolved, and additional molecular detail remains to be elucidated [12]. Similarly, although *MC1R* has been connected to chromosomal stability and centromeric integrity, the mechanism(s) from receptor activation to centromere maintenance remains incompletely defined. Future work will need to determine whether these effects reflect a single coordinated genome-protective program downstream of *MC1R* or several parallel processes that independently reduce melanocyte vulnerability after ultraviolet injury.

### 4.4. Potential Therapeutic Strategies

The possibility that *MC1R*-associated risk might be pharmacologically modified has been explored through several downstream or receptor-directed strategies. One approach is to bypass defective *MC1R* by activating cAMP signaling downstream of the receptor. In a genetically controlled mouse model, D’Orazio and colleagues showed that UV-induced tanning requires intact *MC1R* signaling, but that topical forskolin, an activator of adenylyl cyclase, could restore eumelanin production and prevent UV induced non-melanoma skin cancer formation in *MC1R*-deficient red-blonde mice [13,59].

A related strategy targets signaling downstream of cAMP through salt-inducible kinase (SIK) inhibition. Genetic and pharmacologic studies suggest that SIK signaling constrains CREB-regulated transcriptional coactivators (CTRCs), thereby limiting MITF-dependent pigmentation programs. In particular, SIK2 has been implicated as a negative regulator of MITF and pigment synthesis, while topical small-molecule approaches have generally used inhibitors with activity across multiple SIK isoforms rather than melanocyte-specific SIK2 inhibition [60]. This distinction may be important and benefit from the development of isoform-specific SIK1, SIK2, or SIK3 small-molecule inhibitors.

Small-molecule SIK inhibitors optimized for topical delivery have been shown to increase MITF expression and induce melanogenesis in cultured melanocytes, *MC1R*-deficient mice, and normal human skin ex vivo [14]. The emphasis on topical optimization is clinically important because human epidermis has a substantially greater barrier function than mouse skin, and earlier attempts to translate pigmentation-inducing approaches were limited in part by poor skin penetration of active compounds. SIK inhibition is conceptually attractive because it can activate pigmentation downstream of *MC1R* and may therefore be relevant to individuals carrying red-hair-associated *MC1R* loss-of-function variants. Topical SIK inhibitors have been tested in early human clinical trials where they showed significantly enhanced DNA repair after UV as well as anti-inflammatory effects without alterations in visible pigmentation [61].

A second therapeutic avenue focuses on restoring receptor function itself. *MC1R* palmitoylation, mediated in part by the acyltransferase ZDHHC13, is required for full receptor signaling [15]. In mouse models on a red-haired genetic background, enhancing *MC1R* palmitoylation restored downstream signaling and suppressed melanomagenesis. These studies suggest that *MC1R* loss-of-function is not only a fixed genetic state but also, for at least some variants, a signaling defect that may be partially modifiable [16]. This possibility is encouraging, especially given the broader protective roles attributed to α-MSH and *MC1R* signaling, including effects on pigmentation, oxidative stress, DNA damage responses, and inflammation. However, these interventions remain largely preclinical, and their relevance to melanoma prevention in humans has not yet been established.

While all these possible therapeutic avenues are preclinical, they remain promising opportunities for melanoma treatment and prevention. However, future work is needed to determine which downstream or receptor-directed strategies can produce durable protection in human melanocytes and human skin models. A central question is whether these interventions may be capable of altering only pigmentation or also meaningfully improving oxidative stress resistance, DNA damage repair, genomic stability, inflammatory signaling, and melanoma suppression. Studies using primary human melanocytes and organotypic skin models carrying defined *MC1R* variants would be especially valuable, since red-hair-associated alleles may differ in their residual signaling capacity and responsiveness to rescue.

## 5. Nevi, Diagnostics, and Imaging

### 5.1. Interaction with Nevus Count

Melanocytic nevi (moles) are benign skin lesions that form after *BRAF^V600E^* or *NRAS^Q61^* mutation in melanocytes leads to a period of proliferation followed by oncogene-induced senescence. Increased nevus count is one of the strongest phenotypic predictors of melanoma, and many genetic factors that increase nevus and melanoma risk are linked to cell proliferation and telomere maintenance [62,63]. Typically, nevus numbers increase in more lightly pigmented human skin type individuals. However, interestingly, people with the RHC phenotype have consistently been reported to carry fewer clinically visible nevi than darker-haired individuals, and GWAS studies have shown that *MC1R* variants have either no correlation or inverse correlation with nevus count [63,64,65,66,67]. Landi et al. reported a pleiotropic association for a specific SNP at the *MC1R* locus with nevus-related phenotypes. This SNP effect may reflect variant-specific effects on nevus biology [68]. The explanation for this relationship remains uncertain, but the apparent paradox (between the lightest skin pigmentation and nevus number) may reflect differences in the visibility, pigmentation, or biology of nevi rather than a true absence of melanocytic nevi. In eumelanin-poor skin, some melanocytic lesions may be lighter, flatter, or less clinically conspicuous, raising the possibility that conventional visual nevus counts underestimate the relevant melanocytic burden in this population [22,69]. Selected studies examining the relationship between nevus count, *MC1R* variation, and the RHC phenotype are summarized in Table 1. Studies were included if they reported original human data directly evaluating associations among *MC1R* genotype, RHC-associated pigmentation traits, and nevus number. Studies that did not report nevus-related outcomes or relevant genotype–phenotype associations were excluded. The included studies predominantly evaluated populations of European ancestry or cohorts with lighter pigmentation. Therefore, the applicability of these findings to populations with different *MC1R* allele frequencies, pigmentation phenotypes, and nevus patterns remains uncertain.

This distinction matters because visible nevus count and *MC1R* status capture different dimensions of melanoma susceptibility. High nevus count reflects increased melanocytic proliferation and a greater number of potential precursor lesions, while *MC1R* loss-of-function alters pigment chemistry, ultraviolet sensitivity, oxidative stress, and DNA damage responses. In red-haired individuals, melanoma risk may therefore arise even when the visible nevus count appears modest. Conversely, those with both *MC1R* risk alleles and numerous or atypical nevi represent an especially high-risk subgroup, because potential precursor lesions and pigmentary vulnerability converge [22].

The possibility of clinically subtle or poorly pigmented nevi also has implications for risk stratification. Standard skin examination may underestimate nevus burden in red-haired individuals if lightly pigmented lesions are harder to distinguish from surrounding fair skin or are less likely to be counted as clinically meaningful nevi. Whether clinical examination underestimates nevus burden in red-haired individuals remains an open question. Future studies should compare clinical nevus counts with imaging-derived nevus burden across *MC1R* genotypes, pigmentary phenotypes, and melanoma outcomes [70,71]. These issues make nevus count an important but incomplete marker of melanoma risk in red-haired populations.

### 5.2. *MC1R* Biomarker Informed Risk Stratification and Advanced Imaging

*MC1R* genotype and the RHC phenotype provide complementary but incomplete markers of melanoma susceptibility. *MC1R* variants capture inherited differences in receptor function that may influence melanoma risk beyond visible pigmentation, while red hair, freckling, fair skin, and poor tanning ability remain clinically accessible indicators of pigmentary vulnerability. Neither approach is sufficient alone. Phenotype-based assessment may miss *MC1R* variant carriers without overt red hair, whereas genotype-based assessment does not capture nevus burden, family history, prior melanoma, ultraviolet exposure, tanning behavior, actinic damage, or lesion visibility. Biochemical measures of melanogenesis are conceptually attractive because they lie downstream of *MC1R* signaling, but their clinical role remains uncertain. Although pheomelanin-biased melanogenesis is relevant to red-hair-associated oxidative vulnerability, chemical analyses suggest that epidermal eumelanin and pheomelanin proportions are relatively consistent across skin pigmentation levels, with differences in pigmentation reflecting variation in total melanin content and melanosome distribution [10,72,73,74].

Risk stratification in red-haired or *MC1R* variant populations is likely to be strongest when genotype, visible phenotype, nevus burden, family history, prior melanoma, ultraviolet exposure, and lesion-level clinical features are considered together. Nevus count, atypical nevi, and prior melanoma remain central predictors of risk, but their interpretation may be more difficult in individuals whose melanocytic lesions are lightly pigmented or less conspicuous against fair skin. This supports a prevention strategy based not only on sun avoidance counseling but also on early education, self-examination, structured clinical skin examination, and risk-adapted follow up. Because *MC1R*-associated risk may involve reduced photoprotection, altered pigment chemistry, impaired DNA damage responses, and oxidative stress, surveillance remains relevant even when ultraviolet exposure is minimized.

Imaging approaches differ substantially in their degree of validation and clinical adoption and therefore can be divided into established, emerging, and experimental methods.

Established surveillance methods include dermoscopy, conventional total-body photography, and sequential digital dermoscopic imaging. Total-body photography can help identify new lesions across the skin surface, while sequential digital dermoscopy allows individual lesions to be monitored for subtle structural change over time [75]. These approaches are already used in the longitudinal surveillance of selected high-risk patients, particularly those with numerous or atypical nevi.

Emerging methods include three-dimensional total-body photography and reflectance confocal microscopy. Three-dimensional total-body photography may improve lesion localization and longitudinal comparison, although its incremental benefit over conventional photography remains under evaluation, and broader implementation may increase cost, patient burden, referrals, or excisions [70,76]. Reflectance confocal microscopy can provide near-cellular-resolution assessment of clinically or dermoscopically equivocal lesions, but its use remains concentrated in specialized centers because of cost, operator expertise, and limited availability [77,78].

Experimental or investigational approaches include optical coherence tomography, multispectral and hyperspectral imaging, automated lesion-change detection, and many artificial intelligence-assisted diagnostic systems. Although these methods have shown promise in retrospective datasets, feasibility studies, or selected prospective cohorts, they remain less standardized and less broadly validated than dermoscopy and photographic surveillance [77,78]. AI performance may also be distorted by biases in training datasets. Many image datasets are drawn from specialized centers, contain limited representation of diverse skin tones, and may overrepresent well-pigmented lesions. Amelanotic and hypomelanotic melanomas are particularly important in this context because they are less visually distinctive and may be underrepresented in datasets, potentially reducing algorithmic sensitivity for the lesions in which improved detection is most needed [77,78].

Future studies should test whether adding *MC1R* genotype, RHC phenotype, nevus metrics, lesion pigmentation, and imaging-derived change detection to existing risk models improves screening intervals, biopsy thresholds, and early detection of amelanotic or hypomelanotic melanoma. Such studies should also assess false positive referrals, numbers needed to excise, cost-effectiveness, and patient burden before emerging or experimental technologies are incorporated into routine surveillance.

### 5.3. Amelanotic and Hypomelanotic Melanoma

The diagnostic relevance of the RHC phenotype is not limited to elevated melanoma incidence. It also concerns how melanoma appears on the skin. Amelanotic and hypomelanotic melanomas are associated with the RHC phenotype and are clinically important because they lack some or all of the dark pigment that typically raises suspicion for melanoma [17,79]. Their atypical clinical appearance can contribute to misdiagnosis or delayed recognition. Observational studies have reported poorer survival among patients with clinically amelanotic melanoma. This association may be explained partly by a more advanced stage at diagnosis [80]. Amelanotic lesions contain little visible melanin, while hypomelanotic lesions retain partial pigmentation that may be faint, patchy, or difficult to distinguish from surrounding fair skin. In red-haired and very-fair-skinned individuals, this reduced contrast may make lesions appear pink, red, flesh colored, scar-like, inflammatory, ulcerated, non-healing, or vascular rather than classically melanocytic [21,81]. Studies linking RHC phenotype, *MC1R* variation, and amelanotic or hypomelanotic melanoma are summarized in Table 2. Studies were included if they reported original human data directly evaluating the association of *MC1R* genotype or RHC-associated pigmentation traits with melanoma pigmentation, including amelanotic or hypomelanotic presentation. Studies that discussed *MC1R* biology without reporting lesion pigmentation or relevant clinical associations were excluded. Several findings should be interpreted cautiously because they were derived from small amelanotic or hypomelanotic subgroups. The association reported by Rayner et al. between the *MC1R* R/R genotype and amelanotic or hypomelanotic melanoma was based on only 47 cases and yielded an odds ratio of 26.4 with a wide 95% confidence interval of 6.7–104.7. This should therefore be considered an imprecise exploratory result, as the magnitude of the association remains uncertain. Similarly, the TCGA analysis included only seven amelanotic tumors, all of which carried an *MC1R* variant. Both observations require confirmation in larger, independent cohorts [79,82].

The biology underlying pigment-poor melanoma should not be reduced to a single mechanism. Clinical amelanosis describes how a lesion appears to the patient or clinician, but this appearance can arise from several distinct processes. First, an inherited pigmentation background can reduce visual contrast. A lightly pigmented melanoma on very fair skin may be harder to recognize. Second, tumor-intrinsic defects in melanogenesis may reduce pigment production through loss or downregulation of melanogenic enzymes [82]. Third, melanoma cell-state plasticity can alter pigmentation programs. MITF-high states are generally associated with melanocytic differentiation, pigmentation programs, and proliferation, whereas MITF-low states are associated with dedifferentiation, invasiveness, and alternative transcriptional programs [18,19,83,84,85]. Fourth, therapy-induced dedifferentiation can suppress melanocytic markers and pigmentation in treated tumors, a setting that is biologically distinct from an untreated primary melanoma that appears amelanotic at diagnosis [86,87].

These distinctions are important for interpreting the relationship between *MC1R* status, MITF, and clinical pigmentation. Red-hair-associated *MC1R* variants and other hypopigmentation alleles may predispose a melanoma to a pigment-poor clinical appearance, but this does not mean that all amelanotic or hypomelanotic melanomas in red-haired individuals are MITF-low or fully dedifferentiated [17]. Some pigment-poor melanomas may retain melanocytic lineage markers and MITF expression while producing little visible pigment because of reduced substrate availability, impaired melanogenic enzyme activity, or altered melanosome biology. Conversely, some truly amelanotic tumors may reflect tumor-intrinsic loss of melanocytic differentiation, deletion/silencing of key pigment enzymes (such as tyrosinase), or therapy-induced dedifferentiation. Future studies should therefore evaluate *MC1R* genotype, inherited pigmentation background, MITF expression, melanogenic enzyme activity, tumor pigmentation, treatment history, dermoscopic and confocal features, and clinical detectability in the same lesions, rather than assuming a one-to-one relationship between *MC1R* variation, MITF state, and amelanotic appearance.

## 6. Conclusions and Future Directions

Red-haired populations occupy a distinctive position in melanoma biology, risk prediction, and diagnosis. Their elevated melanoma susceptibility is not explained by light skin or reduced ultraviolet shielding alone. Loss-of-function variation in *MC1R* is associated with diminished cutaneous eumelanin, thereby reducing eumelanin-associated protection and redox buffering while favoring a pheomelanin-rich state associated with oxidative stress. Diminished *MC1R* signaling also appears to impair DNA damage responses and genome maintenance [10,12,42,72,88]. Together, these findings suggest that the RHC phenotype marks a broader biological state of melanoma vulnerability.

Several mechanistic questions remain unresolved. The chemical basis of pheomelanin-associated oxidative stress in intact human skin requires further clarification, particularly the relative contributions of ultraviolet absorption, antioxidant depletion, cysteine consumption, reactive oxygen species generation, and melanosomal redox chemistry. Similarly, although *MC1R* signaling has been linked to nucleotide excision repair, chromosomal stability, and centromeric integrity, the downstream steps precisely connecting receptor activation to these genome protective processes remain incompletely defined [11,12].

The possibility that *MC1R*-associated risk may be pharmacologically modifiable also deserves further study. These interventions remain pre-clinical, and future experiments should test whether such interventions restore not only pigmentation but also oxidative stress resistance, DNA repair capacity, genomic stability, inflammatory regulation, and melanoma suppression in primary human melanocytes and organotypic skin models carrying defined *MC1R* variants.

These biological uncertainties have direct implications for risk stratification. Red hair, freckling, poor tanning ability, *MC1R* genotype, nevus burden, family history, prior melanoma, and ultraviolet exposure can identify individuals whose melanoma risk is elevated before disease develops, but no single marker fully captures susceptibility. Visible phenotype is an incomplete proxy for genotype, and genotype alone does not capture nevus burden, ultraviolet exposure, family history, prior melanoma, or lesion visibility [20,22,42]. Biochemical measures of melanogenesis, including total melanin content and melanin degradation products, may help connect inherited pigmentation biology to pigment chemistry in research settings, but they are not yet validated for routine melanoma susceptibility prediction. Future models should evaluate whether combining *MC1R* genotype, visible phenotype, nevus burden, family history, prior melanoma, ultraviolet exposure, pigment biology, and imaging-derived lesion metrics improves melanoma detection, surveillance intervals, and biopsy decisions beyond standard phenotype-based approaches.

A major unresolved diagnostic question is whether eumelanin pigment-poor melanoma in red-haired populations reflects reduced visual contrast, inherited pigmentation background, altered melanogenesis within the tumor, melanoma cell-state switching, or a combination of these processes. MITF provides one framework for studying this issue, since MITF-high states are generally associated with melanocytic differentiation and pigmentation programs, whereas MITF-low states are associated with dedifferentiation and invasiveness [83,85]. However, eumelanin pigment-poor melanoma should not be assumed to be uniformly MITF-low. Some amelanotic or hypomelanotic melanomas may retain melanocytic lineage markers while producing little visible pigment because of inherited or somatic defects in melanogenesis or altered melanosome biology [18]. Future studies should evaluate *MC1R* genotype, inherited pigmentation background, MITF expression, melanogenic enzyme expression, tumor pigmentation, treatment history, dermoscopic appearance, confocal or other imaging features, and clinical detectability in the same primary lesions.

A more complete approach to melanoma prevention and early detection in red-haired populations will require integration across molecular biology, risk prediction, imaging, and clinical surveillance. *MC1R*-informed risk prediction should be considered promising but not yet clinically settled. By linking *MC1R* biology to both melanoma susceptibility and melanoma recognition, future work can move toward prevention and diagnostic strategies tailored to the distinctive vulnerabilities of red-haired populations.

## Figures and Tables

**Table 1:** *MC1R* and RHC phenotype and melanocytic nevus count across clinical and genome-wide studies.

Study	Year	Study Type	Population/*n*	Effect Measure	Finding for Nevus Count	Limitations	Ref
English & Armstrong	1994	Clinical (cross-sectional)	Australian schoolchildren ~6 yr, *n* = 511	Nevus count by hair color	Red hair is associated with lower nevus counts than other hair colors, while fair, sun sensitive traits were positively associated with nevi.	Nevus counts were not directly validated, and the low, potentially selective participation rate may have inflated nevus prevalence and biased associations with demographic factors.	[65]
Synnerstad et al.	2004	Clinical (cross-sectional)	Swedish children 8–9 yr, *n* = 524	Median nevi count by pigment trait	Fair skinned and light eyed children had significantly more nevi, whereas red-haired children had very few.	Nevi smaller than 2 mm were excluded to reduce misclassification with freckles, and inter and intrarater reliability of nevus counts was not formally assessed.	[67]
Dellavalle et al.	2005	Clinical (cohort)	U.S. (Colorado) 3-yr-olds, *n* = 280	Nevus count by hair color	Red-haired children had more freckles but fewer melanocytic nevi than non-red haired children.	Small cohort of 280 three-year-old children from Colorado. young age, regional sampling, and few red-haired participants	[64]
Duffy et al.	2018	GWAS (nevus + melanoma pleiotropy)	Nevus meta-analysis *n* = 52,506; melanoma 12,874 cases/23,203 controls	Per-allele OR (melanoma) vs. nevus count association	*MC1R* variants show no association with nevus count	Heterogeneous nevus count methods and between study variation in SNP associations, potentially related to differences in age and sun exposure across cohorts.	[63]
Landi et al.	2020	GWAS (melanoma meta-analysis, 54 loci)	*n =* 36,760 melanoma cases/375,188 controls	Per-allele OR, melanoma	*MC1R* region SNP pleiotropically associated with nevus counts.	European ancestry only. The *MC1R* region GWAS signal does not establish a causal variant or mechanism for nevus count.	[68]
Jayasinghe et al.	2026	GWAS (nevus-count meta-analysis, 14 studies)	n = 85,965 (European ancestry); 29 nevus loci	Per-allele effect on nevus count; pathway enrichment	*MC1R* variants are associated with reduced nevus count	European ancestry only. Heterogeneous nevus phenotyping across cohorts, including self reported and regional counts.	[66]

**Table 2:** *MC1R* variant status and red-hair phenotype in amelanotic and hypomelanotic melanoma.

Study	Year	Cohort/Design	n (AHM vs. Comparator)	*MC1R*/Red-Hair Association with AHM, OR (95% CI)	Limitations	Ref
Vernali et al. (GEM)	2017	GEM. International, population/hospital-based. Incident primary cutaneous melanoma. Logistic regression	178 amelanotic vs. 2209 pigmented (of 2387)	*MC1R* variant carriage (r/r, R/r, or R/R vs. wt/wt) associated with amelanotic melanoma: OR 1.70 (1.04–2.78); attenuates to 1.43 (0.86–2.39) after phenotype adjustment.	Possible observer variation in classifying tumors as amelanotic or pigmented.	[17]
Wee et al.	2018	Victorian Melanoma Service. Prospective cohort; clinically amelanotic/hypomelanotic vs. pigmented. Multivariable logistic regression	384 AHM vs. 3529 pigmented (9.8% of 3913)	Red hair independently associated with AHM: OR 2.0 (1.2–3.2) adjusted.	Single center cohort with potentially subjective clinical pigmentation classification. *MC1R* genotype was not evaluated.	[19]
Strazzulla et al.	2019	Single tertiary-center retrospective record review. Clinically amelanotic (AMM) vs. pigmented (PMM)	342 AMM vs. 591 PMM (933 total)	Red hair associated with amelanotic melanoma: OR 1.81 (1.02–3.21).	Retrospective review of records from a single center, with possible missing or inconsistently documented data and limited generalizability.	[80]
Rayner et al. (BNMS)	2019	Brisbane Naevus Morphology Study. Case–control. AHM vs. pigmented melanoma vs. controls.	47 AHM vs. 389 pigmented (of 436). 692 controls	*MC1R* R/R genotype strongly associated with AHM: OR 26.4 (6.7–104.7) vs. wt/wt.	Small amelanotic/hypomelanotic subgroup produced an imprecise estimate with a wide confidence interval	[79]
Rayner et al. (BNMS)	2020	Brisbane Naevus Morphology Study + TCGA SKCM. Whole-exome sequencing + microarray. AHM vs. pigmented melanoma vs. controls.	45 AHM vs. 389 PM (BNMS). 7 AHM vs. 463 PM (TCGA)	All 7 TCGA amelanotic tumors carried an *MC1R* variant. None were wild-type/wild-type.	The TCGA amelanotic subgroup included only seven tumors, limiting precision and generalizability. The observed universal *MC1R* variant carriage requires confirmation in a larger independent cohort.	[82]

## Data Availability

No new data were created or analyzed in this study. Data sharing is not applicable to this article.

## Abbreviations

The following abbreviations are used in this manuscript:

*MC1R*	Melanocortin 1 Receptor
ROS	Reactive Oxygen Species
CPD	Cyclobutane Pyrimidine Dimer
SIK	Salt Inducible Kinase
RHC	Red Hair Color
PKA	Protein Kinase A
ASIP	Agouti Signaling Protein
